# Long‐term performance of show‐jumping horses and relationship with severity of ataxia and complications associated with myeloencephalopathy caused by equine herpes virus‐1

**DOI:** 10.1111/jvim.17070

**Published:** 2024-04-12

**Authors:** María de la Cuesta‐Torrado, Ana Velloso Alvarez, Patricia Neira‐Egea, Juan Cuervo‐Arango

**Affiliations:** ^1^ Department of Animal Medicine and Surgery Universidad CEU‐Cardenal Herrera, CEU Universities Valencia Spain; ^2^ Veterinary Teaching Hospital, Universidad Cardenal Herrera CEU Valencia Spain

**Keywords:** EHM, neurologic, outbreak, sport horse

## Abstract

**Background:**

Equine herpesvirus myeloencephalopathy (EHM) has severe impact on the sport horse population.

**Objective:**

Study the influence of EHM on the likelihood of affected horses to return to their previous performance and investigate the association of clinical variables with prognosis.

**Animals:**

Twenty‐six horses positive for equine herpesvirus type 1 (EHV‐1) were admitted to a veterinary teaching hospital (VTH) during a natural EHM outbreak at an international jumping event.

**Methods:**

Data collected from the VTH, the International Equestrian Federation, and surveys completed by the riders and horse owners were retrospectively analyzed.

**Results:**

Horses affected by EHM had 68% chance of returning to exercise, and 52.9% were able to achieve their preoutbreak performance level. Horses with an ataxia grade at admission ≥4/5 had an increased fatality rate (*P* < .05) and 10% chance of reaching their preoutbreak performance level. None of the horses with both vascular and urinary complications returned to their previous performance level. Finally, horses vaccinated against EHV‐1 and those with urinary complications had a 71.4% and 43.7% fatality rate, respectively.

**Conclusions and Clinical Importance:**

Horses affected by EHM were able to return to their previous performance levels, but certain clinical variables were negatively associated with postoutbreak performance. Ataxia grade upon admission and the development of systemic signs of vasculitis and urinary complications were potential poor prognostic indicators in sport horses. Variables linked to fatality included prior vaccination against EHV‐1, ataxia grade upon admission, and the development of urinary complications.

**Abbreviations**
CTL: cytotoxic T‐lymphocytes
EHM: equine herpesvirus myeloencephalopathy
EHV: equine herpesvirus
EMS: muscle electrostimulation
FEI: International Equestrian Federation
MLST: multilocus sequence typing
VTH: veterinary teaching hospital

## 1 INTRODUCTION

Equine herpesvirus type 1 (EHV‐1) is ubiquitous in horse populations worldwide, and many horses are latently infected.^1^ Equine herpesvirus‐1 can lead to equine herpesvirus myeloencephalopathy (EHM), which is a complex disease of horses. It is considered a complication after EHV‐1 respiratory tract infection and viremia,^1^ where severe damage to nervous tissue occurs because of an acute inflammatory process involving vasculitis, thrombosis, and ischemia.^2^ Infections caused by EHV‐1 are common in young performance horses within the first weeks or months of life. This infection typically results in the establishment of latent infection with subsequent viral reactivation causing clinical disease and viral shedding during periods of stress.^3^ The estimated prevalence of latent infection ranges between 54% and 88%.^4^

Viremia is a common consequence of EHV‐1 infection. Approximately 10% of infected horses develop neurological signs during EHM outbreaks.^5^ Currently, 3 genotypes of EHV‐1 have been identified in EHV‐1 infections: nonneuropathogenic (N752), neuropathogenic (D752), and a new variant (H752).^6^ The prognosis of EHM patients that are recumbent or need support from slings is considered unfavorable.^7^ In recent years, an increase in the incidence of EHM outbreaks in different geographical areas has been reported in France,^8^ Netherlands,^5^ and the United States.^9^, ^10^, ^11^

Sport horses are at increased risk of being involved in an outbreak of EHV‐1. The large number of competing horses from different regions, increased movement of horses during the shows, transport‐related stress, and temporary housing with decreased ventilation are some of the variables that potentially put these animals at risk of infection.^3^, ^11^ Numerous studies have investigated the association of different risk factors with the development, severity, and prognosis of EHM in horses during an EHV‐1 outbreak.^2^, ^9^ However, limited information is available on the relationship between the presence and severity of EHM and the likelihood of affected horses returning to exercise as a long‐term outcome in show‐jumping horses. New information regarding EHV‐1 infected horses, such as identification of EHM clinical variables associated with poor prognosis of returning to preoutbreak performance level, would be relevant in decision‐making for horse owners and veterinarians during an EHV‐1 neurological outbreak, because EHM often raises important welfare and economic concerns in high‐performance horses. Our main objective was to determine the relationship between the development and severity of EHM and the long‐term likelihood of affected horses returning to previous sport performance levels. A secondary objective was to determine the association between clinical variables observed during admission and progression of the disease, and their impact on long‐term performance level.

## 2 MATERIALS AND METHODS

Patient data were obtained from hospitalized horses in the veterinary teaching hospital (VTH), after the EHV‐1 outbreak that occurred in Valencia, Spain, in February 2021 during a show jumping international competition event (International Equestrian Federation [FEI] approved).

### 2.1 Description and history of the outbreak

On February 20, 2021, the first patient was admitted to the veterinary teaching hospital (VTH) with clinical signs of pyrexia and grade ataxia 2/5.^12^ The patient was stabilized and discharged 6 days later. Because of the risk of a possible EHV‐1 outbreak, the competition authority began monitoring horses for pyrexia (rectal temperature ≥ 101 °F) twice a day, as well as for any neurological signs. Over the subsequent days, an increase in the number of horses presented for pyrexia and ataxia was noted. The next day the FEI Veterinary Department was notified of 20 febrile horses at the competition and 1 horse that had recently returned to France after participation in the event was confirmed positive for EHV‐1 and presented with neurological signs. Subsequently, the show‐jumping event was canceled.

On February 25th, the next patient was admitted with neurological signs of grade 4/5 ataxia and altered mental status, displaying clinical signs of stupor and aggressiveness. Within the next 24 hours, many more cases were detected in the competition facilities, with affected horses developing pyrexia, ataxia, urinary incontinence, or some combination of these signs. Affected horses were promptly referred to the VTH, and EHV‐1 patients were hospitalized in the isolation area. Because of the severity of the outbreak and high risk of contagion, VTH management made the decision to discharge all other horses in the hospital unrelated to the EHV‐1 outbreak except a teaching mare owned by the University, that was in the hospital for treatment of a corneal ulcer. On February 28th, the resident mare owned by the University began to exhibit pyrexia without other associated clinical signs. A nasal swab and blood sample were taken for EHV‐1 testing. Because of the highly contagious nature of the virus, it was assumed that the mare was a probable positive case. Given the high number of horses affected with severe neurological signs at that time in the competition, the hospital was closed so as to exclusively manage the EHV‐1 outbreak cases.

The decision to refer horses to the VTH was based on clinical criteria of disease severity, agreed between the veterinarians at the competition and the equine clinical service of the VTH. Patients with a grade of ataxia ≥3/5 according to a previously published modified scale,^12^ altered mental status and cranial nerve dysfunction were prioritized for referral to the VTH. Additionally, horses with lower ataxia grades (0‐2) but having difficulty urinating and requiring indwelling urinary catheterization or those with signs of colic also were included. By the end of that week, from a total of 160 horses held at the competition facilities, 118 were found to be clinically or subclinically infected with EHV‐1,^13^ and 25 horses were referred to the VTH.

### 2.2 Animals admitted to the VTH

Of the 25 horses referred to the VTH, 24 horses were Central European breeds: Holsteiner, Oldenburg and Koninklijk Warmbloed Paardenstamboek Nederland, and 1 Spanish horse. Sixteen horses were females, and 9 were geldings. The mean age was 9.7 ± 2.3 years old (range, 6‐15 years). The origin of the animals was Germany (n = 10), Switzerland (n = 3), Belgium (n = 5), Sweden (n = 1), France (n = 3), and Spain (n = 3). The teaching mare resident at the VTH was a crossbred 10 years of age.

Of the 25 admitted horses, 18 horses had an ataxia grade ≥3/5 upon arrival. The remaining horses (n = 7) showed lower ataxia grade (≤2/5) but also had clinical signs such as alterations in mental status, difficulty urinating, or colic. Upon admission, a thorough history was taken for each patient, compiling information provided by the competition's veterinarians and horse owners. This information included: breed, sex, age, vaccination status upon arrival at the show jumping event, specific clinical signs observed, previous treatments received, days of viremia (from the onset of pyrexia), and days from identification of first neurological signs. Day 1 of disease was considered the first day on which the horse had pyrexia (rectal temperature ≥101 °F).

### 2.3 Vaccination status of referred horses

Vaccination records for the year before the outbreak were obtained for all horses by checking the patients' passport vaccination records. An appropriate vaccination status was considered to be 2 initial first doses of EHV‐1 vaccine at a 4‐week interval, followed by at least 1 booster yearly, whereas horses with no record of vaccination against EHV‐1 in their passport were considered unvaccinated.

### 2.4 Diagnosis and therapeutic management of EHV‐1 affected horses

Upon admission, a blood sample was taken from every patient to perform a complete blood analysis. Nasopharyngeal swabs and blood samples in ethylenediaminetetraacetic acid (EDTA) tubes were collected for PCR. Samples then were sent to the national official laboratory in Spain. Regardless of the results, all patients admitted with clinical signs of pyrexia or neurological sign were considered positive for EHV‐1, because they originated from the initial outbreak location. A laboratory in Germany subsequently performed viral sequencing.

Stall confinement was initiated during the first week of the outbreak for patients with ataxia grades ≥3/5, whereas those with ataxia grades <3/5 were prescribed three 10‐minute walks per day. The horses were physically examined in their stables at least 6 times per day. A neurological examination was performed daily on all horses to adapt treatment to each patient's progression, according to the recommendations established for EHM.^3^ A detailed description of the medical treatments and therapeutic management of the affected horses can be found in Supplementary Information S1.

### 2.5 Clinical signs and associated complications on EHV‐1 patients

Possible associations between the severity of disease and outcome were analyzed. Clinical signs and associated complications, other than pyrexia and ataxia, were classified into 3 categories: (1) clinical signs of vasculitis (patients developing ≥1 of the following findings, which were identified by the clinician during physical examination: limb edema, myocarditis, thrombi, petechiae or some combination of these; (2) urinary problems (urinary incontinence and cystitis); and, (3) other complications (musculoskeletal, colic, or ophthalmological problems). Additional information can be found in Supplementary Information S2. The treatment (medical or surgical) of horses with clinical complications was performed according to the specific in each case. Patients with ataxia grade ≥4/5 were supported using a sling for several days. Euthanasia was performed for patients exhibiting advanced neurological alterations unresponsive to treatment or experiencing serious systemic complications resulting from the disease, after informed consent from the owners.

### 2.6 Physical therapy

Once the patients recovered from the critical phase of the disease, rehabilitation exercises were initiated. This therapy involved proprioception exercises, core work, and neuromuscular electrical stimulation. A detailed description of the physical therapy can be found in Supplementary Information S3.

### 2.7 Follow‐up after discharge from the hospital and long‐term outcome

#### 2.7.1 Follow‐up owner questionnaire

**FIGURE 1:**
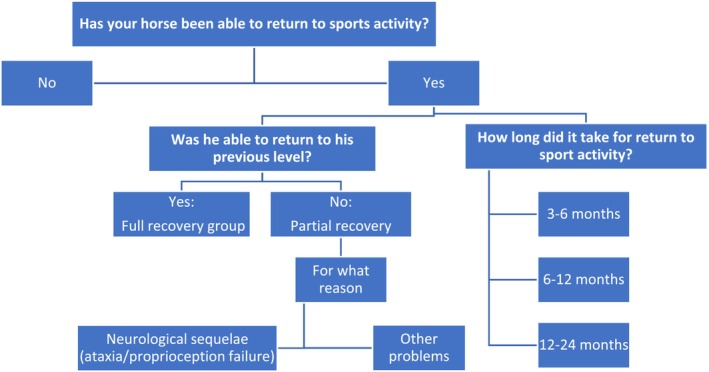
Diagram of survey information.

A telephone survey was conducted with the owners or riders 2 years after their horses´ admission to the VTH to collect feedback on their horse's performance in the 2 years after EHV‐1 infection (Figure 1). Based on these data, each horse was given a survey score: survey score of 1 was given to horses that had returned to their previous sport level of performance whereas a survey score of −1 was given to those horses that could not reach their prior performance level or did not return to exercise.

#### 2.7.2 Long‐term outcome

Long‐term outcome also was evaluated by comparing the jumping performance level 2 years before EHV‐1 infection and 2 years after recovery, depending on the time of return to competition. Data were collected from the FEI website for each horse, in the section https://Data.fei.org/horse, and 3 variables related to the performance of jumping horses were analyzed^14^, ^15^, ^16^: (1) average height of the jumped obstacle for each competition; (2) mean value of the final position in the tests performed (ranking); and (3) number of competition entries in a given period of time. Based on these data, each horse was given a score, termed the FEI Score: score FEI = 1, when the horse had the same or improved mean score in at least 2 of the 3 variables analyzed: similar (*P* > .05) or increased (*P* < .05) mean number of competition entries during the studied period, similar (*P* > .05) or increased (*P* < .05) mean height (cm) of the obstacles, or similar (*P* > .05) or decreased (*P* < .05) ranking score (a lower score implies a better classification). On the contrary, horses were given a score FEI = −1 when records showed poorer results in at least 2 of the 3 variables: decreased (*P* < .05) mean number of competition entries, decreased (*P* < .05) mean height (cm) of the obstacles, and increased (*P* < .05) ranking score.

A total score was given for each horse after adding the values of the FEI and survey scores. Horses with a score ≥1 were included in the full recovery group, whereas horses with a score <1 were included in the partial recovery group.

### 2.8 Statistical analyses

Data collected from the horses included in the study (sex, age, breed, presence of neurological signs, EHV‐1 vaccination status, and presence of complications) were evaluated using descriptive statistics. Continuous data were tested for normality using the Anderson‐Darling test. Data not normally distributed were analyzed by nonparametric tests. For statistical analysis, the onset of pyrexia to EHM intervals was categorized into 2 groups: (1) horses with short interval (1‐3 days) and (2) horses with longer interval (6‐7 days). A Fisher's exact test was used to determine the association between the fatality rate at VTH discharge and the following clinical variables: vaccination status, interval from onset of pyrexia to EHM, development of systemic signs of vasculitis, urinary complications, or other complications. The relationship between ataxia grade on admission and the development of urinary complications and systemic signs of vasculitis during the progress of disease was analyzed using binary logistic regression. Differences in the performance level (according to FEI data) between the pre‐ and post‐EHV‐1 outbreak periods for each horse were analyzed using a Wilcoxon signed rank test. Finally, the difference in the ataxia grade on admission of horses that were euthanized or partially or fully recovered was tested using a Kruskal‐Wallis nonparametric test. All of the analyses were performed using commercially available software (IBM SPSS Statistics software version 20) with a *P*‐value for significance set at <.05.

## 3 RESULTS

Twenty‐six horses were treated in the VTH during the outbreak, and 20 horses were discharged from the hospital. During hospitalization, 7 horses were euthanized for different reasons: severe central nervous system signs after colic surgery (n = 1), uroperitoneum (n = 2), comatose state unresponsive to treatment within hours of being admitted to the VTH (n = 4), and 1 horse was euthanized 72 hours after admission because of worsening of neurologic signs unresponsive to treatment. Two months after hospital discharge, 1 horse was euthanized because of severe laminitis. The fatality rate at the time of discharge was 26.9%, whereas the fatality rate of all horses admitted to the VTH was 30.8% (including the horse euthanized after discharge). Viral sequencing analysis carried out by an external laboratory, identified the N variant (point mutation EHV‐1 A2254), using multilocus sequence testing (MLST) for EHV‐1. Results for nasopharyngeal swabs and blood samples analyzed by PCR were 23/25 (92%) and 19/25 (76%), respectively, 17/25 (68%) were positive and 1/25 (4%) resulted in negative results for both samples. One patient could not be tested because of rapid deterioration, which resulted in euthanasia.

**FIGURE 2:**
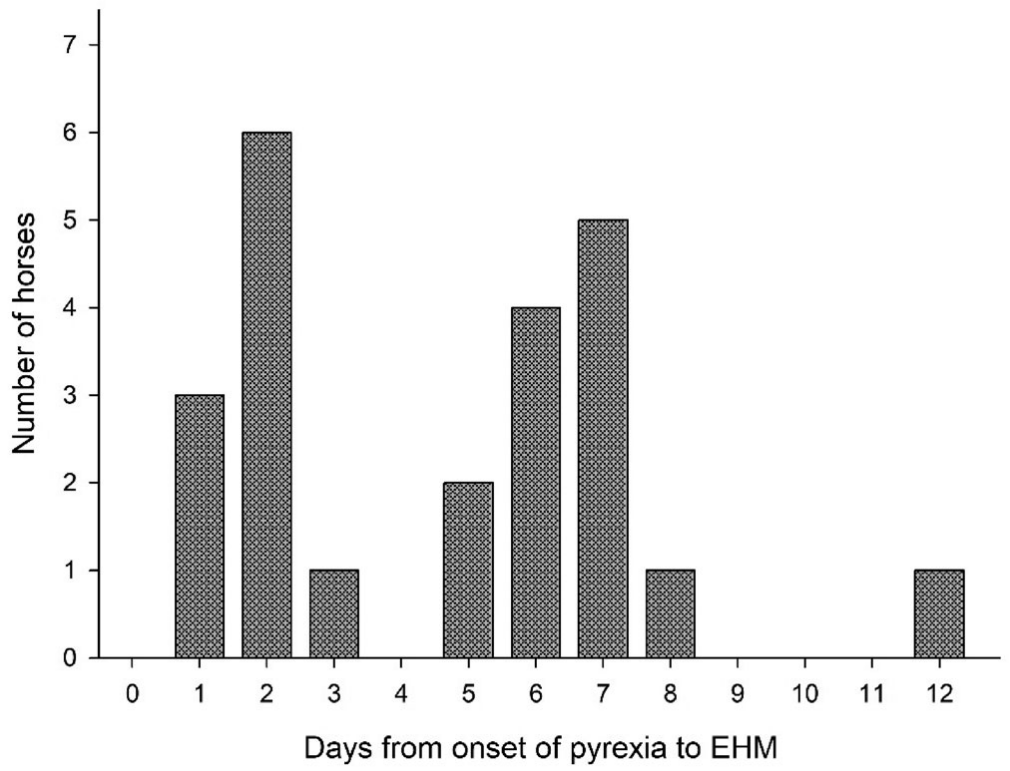
Histogram distribution of the interval from the onset of pyrexia to the development of neurological signs. Horses with a shorter interval (1‐3 days), between the onset of pyrexia to the development of EHM, presented a fatality rate of 20% (2/10) compared with 56% in horses with an interval of 6 to 7 days (5/9; *P* = .1). EHM, equine herpesvirus myeloencephalopathy.

The distribution of the interval between the onset of pyrexia and development of EHM showed a biphasic pattern (Figure. 2). Horses with a shorter interval (1‐3 days) had a fatality rate of 20% (2/10) compared with 56% in horses with an interval of 6 to 7 days (5/9; *P* = .1).

All but 1 horse had at least 1 type of complication: 17/26 (65%) showed systemic signs of vasculitis: 4 limb edema, 1 myocarditis, 3 thrombophlebitis, and 12 petechiae (in the oral or vulvar mucous membranes); 16/26 (61%) had urinary system signs: 2 uroabdomen; 16/26 incontinence and cystitis; and, in the group of other complications, 7/26 (26.9%) horses developed clinical signs of colic, 2/26 (7.7%) horses showed ophthalmologic complications, and 1 horse developed laminitis. A cardiovascular examination was conducted in the patient with presumed myocarditis on arrival because of sustained tachycardia: paroxysmal polymorphic ventricular tachycardia and frequent premature ventricular beats were found on ECG, dyskinesia during systole in the basal septum was found on echocardiography, and a clinically relevant increase in serum concentration of troponin I was identified. After corticosteroid treatment, clinical findings improved. Ophthalmological concerns consisted of sudden‐onset superficial ulcers that resolved without complications. After discharge from the hospital, 2 horses had long‐term complications: 1 horse developed exercise‐induced pulmonary hemorrhage, whereas the other horse developed headshaking.

**FIGURE 3:**
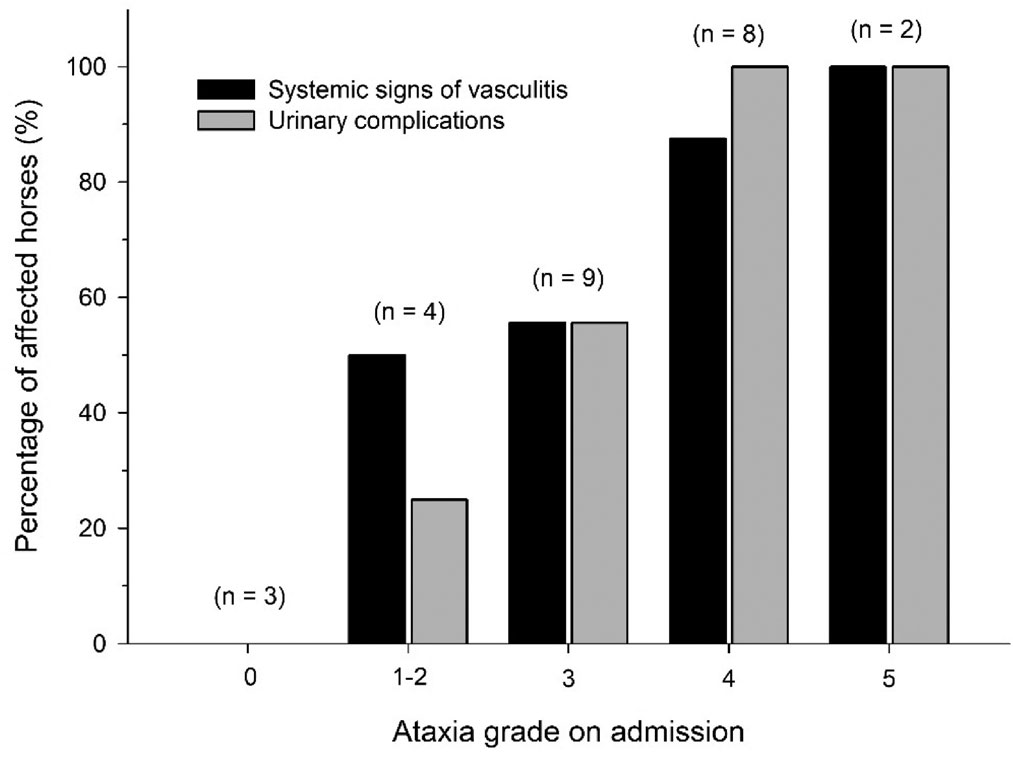
Relationship between the presence of systemic signs of vasculitis/urinary complications and the ataxia grade on admission. Horses with higher grade of ataxia on admission were more likely to develop urinary complications (*P* = .03) or show systemic signs of vasculitis (*P* = .06).

The main clinical variables associated with survival at the time of discharge are shown in Table 1. Vaccinated horses were more likely (*P* = .01) to be euthanized (5/7, 71.4%) than unvaccinated horses (2/19, 10.5%). The development of urinary complications was associated with increased fatality rate (*P* = .02; Table 1). Ataxia grade on admission was associated with increased likelihood of developing urinary complications (*P* = .03; odds ratio [OR] = 0.12) or showing systemic signs of vasculitis (*P* = .06; OR = 0.54; Figure 3). Furthermore, horses with an ataxia grade on admission ≥4/5 (n = 10) were more likely to develop urinary complications (10/10, 100%; *P* = .003) and showed systemic signs of vasculitis more frequently (9/10, 90%; *P* = .08) than horses with ataxia grade ≤3/5 (6/16, 37.5% and 8/16, 50%, respectively). The remainder of complications (musculoskeletal, colic, or ophthalmological) were not associated (*P* > .1) with the degree of ataxia on admission or the fatality rate. Although lymphopenia (22/26, 84%) was the most common abnormal finding on the admission CBC, no association was found with survival or the grade of ataxia on admission (*P* > .1).

**TABLE 1:** Association between vaccination status before the outbreak and development of complications during the progress of disease on the likelihood of survival at discharge.

	n	Preoutbreak vaccination		Urinary complications		Systemic signs of vasculitis		Urinary + vasculitis	
		Yes	No	Yes	No	Yes	No	Yes	No
Hospitalized	26	7	19	16	10	17	9	11	15
Survived	18	2	17	9	10	11	8	5	14
Euthanized (%)	7 (26.9)	5 (71.4)	2 (10.5)	7 (43.7)	0 (0.0)	6 (35.2)	1 (11.1)	6 (54.5)	1 (7.1)
*P* value		.01		.02		>.1		.02	

Variations in sport performance (“total score”) between the pre‐ and postoutbreak periods for individual horses based on FEI and owners and riders survey data can be found in Supplementary Table 1 in Supplementary Information S4. Based on the total score classification from the 17 horses that survived the disease, 47.1% (8/17) were unable to regain their preoutbreak level of performance (partial recovery). On the other hand, the remainder of horses (9/17, 52.9%) did achieve their preoutbreak level of performance (full recovery). Results from the telephone survey showed that all horses (n = 17 responders) were able to return to training and attain some level of performance within 3 and 24 months after discharge from the hospital (2/17, 3‐6 months; 12/17, 6‐12 months; and 3/17, 12‐24 months). However, only 15 horses returned to FEI competitions, whereas 2 horses retired from competition because of sequelae from the disease (residual ataxia). These 2 animals were used for breeding. The horse euthanized 2 months after being discharged because of severe laminitis was not included in the sport performance analyses, because it was categorized within the fatality group.

**FIGURE 4:**
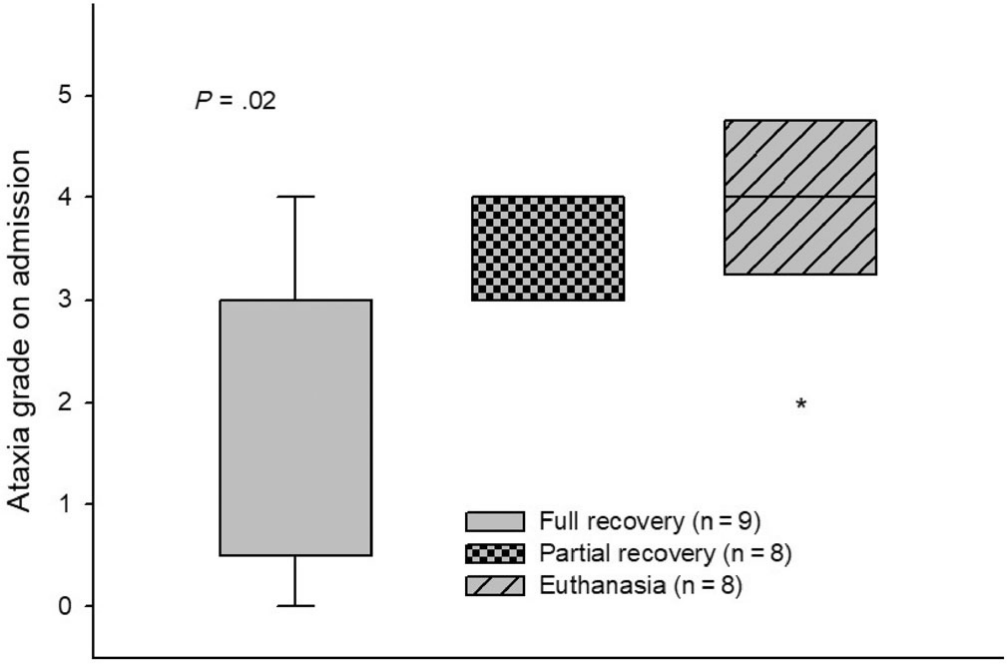
Box plot distribution of ataxia grade on admission of horses with different outcomes. Full recovery: horses that returned to exercise and reached a similar or improved preoutbreak sport performance based on the score obtained from FEI and survey data. Partial recovery: horses that returned to exercise but did not reach the same preoutbreak sport performance based on the score obtained from FEI and survey data. On the first box, the whiskers in the y axis show maxim and minimum ataxia grades in the patients of full recovery group, in the other two groups all values are within the interquartile range, except one outlier (*) in the euthanasia group. The median ataxia score on admission differed significantly (*P* = .02) among different groups (horses that were euthanized or recovered partially or fully). FEI, International Equestrian Federation.

Ataxia grade upon admission was negatively associated (*P* = .02; Figure. 4) with the outcome of the disease. Patients with ataxia ≥4/5 (n = 10) had a 60% chance of being euthanized, 30% chance of returning to exercise but without reaching preoutbreak performance level (partial recovery), and only 10% chance of returning to preoutbreak performance level (full recovery). On the other hand, the outcome of patients with ataxia ≤3/5 (n = 15) was more favorable (*P* = .02): 13.3% were euthanized (n = 2), 40% recovered partially (n = 6), and 46.6% (n = 7) recovered fully and were able to compete again at the same preoutbreak performance level. In addition, horses that fully recovered did not develop any of the 2 complications simultaneously (urinary complications and systemic signs of vasculitis) during the EHV‐1 infection (0/9, 0%), whereas those that showed both complications were more likely to be included in the partial recovery group: (5/8, 62.5%; *P* = .01).

## 4 DISCUSSION

Ours is the first study to report the likelihood of long‐term return to exercise and jumping performance level in FEI competitions of horses recovered from a neurological outbreak of EHV‐1. Horses with EHM had a good prognosis for returning to some level of exercise (68% of horses admitted to the VTH were able to jump again). However, not all horses returned to their previous performance level. Only 52.9% of horses discharged from the hospital were able to reach a similar or improved preoutbreak level of performance. It is important to emphasize the negative association found between ataxia grade, development of complications (urinary and vascular), and likelihood of full recovery. Horses that had an ataxia grade ≥3/5 upon admission experienced a notable decrease in the likelihood of full recovery, whereas horses graded ≥4/5 showed minimal likelihood for achieving full recovery. Most horses underwent active physical rehabilitation from the stable phase of the disease during the outbreak, which also could have a positive impact on the neuromuscular recovery of horses and sports prognosis.^17^

In addition to the grade of ataxia on admission, the development of complications, especially in the vascular and urinary systems, had a relevant impact on survival and prognosis of the affected horses. The presence of these complications could be secondary to a higher degree of viremia and even possible replication of the virus in locations not previously described, such as the urinary system. Lesions in the bladder mucosa in cases that have not undergone urinary catheterization have been documented previously.^18^ Furthermore, human alpha‐herpesvirus 1 has been implicated in a subset of human patients with gastric ulceration and viral replication in urinary tissue.^19^ A recent study has shown the detection of EHV‐1 in equine urine.^20^ Future studies are necessary to determine if the bladder lesions are caused by catheterization or viral replication in the bladder mucosa. Horses naturally infected with EHV‐1 had not been reported to develop myocarditis associated with EHM until recently. A 2023 study analyzing some of the epidemiological data from the Valencia outbreak of 2021^21^ described the case of 1 EHV‐1 infected horse suffering from myocarditis that was attended by the hospital team of our present study. This horse had prolonged viremia and did not recover fully, which is further evidence of the association between vascular complications and poor prognosis.^22^ With regard to other long‐term complications in our study, headshaking also is reported in the literature as a complication of EHV‐1 infection and is related to the presence of latent infection in the trigeminal ganglia.^23^ However, the described complications also may occur independent of the disease. Therefore, the relationship of the virus with these complications should be interpreted with caution. Additional studies are needed before a causal relationship between EHV‐1 infection and these complications can be confirmed.

The prognosis and probability of return to jumping of EVH‐1 infected horses is a very frequent question among horse owners in an EHV‐1 outbreak, because the investment of time and economic resources for these types of patients is usually very high. Therefore, it is important to make appropriate decisions according to the likelihood of full recovery.^4^, ^24^ Our results showed that a high ataxia grade (≥4/5) and development of urinary and vascular complications might be useful indicators of poor performance prognosis, because none of the horses showing urinary complications and systemic signs of vasculitis simultaneously and only 10% of horses with ataxia grade ≥4/5 on admission returned to the preoutbreak performance level.

Evaluating the performance of horses in FEI competitions is challenging because of various factors that influence outcome including rider, surface, as well as height, type and number of obstacles, and quality of other competitors or environmental conditions.^14^, ^15^, ^16^, ^25^ Surveys conducted with owners, riders, or officiating staff are a valuable tool, often providing the only available data for the researcher. Such information has been used as a basis for retrospective descriptive studies of the disease,^26^ but often can be influenced by the subjectivity of those interviewed and may not be entirely reliable.^27^ The subjectivity of assessing the performance level of jumping horses is a possible limitation of our study, because it can be difficult and variable. Therefore, to minimize the subjectivity of this assessment, a combination of 2 evaluation methods for assessing horse performance was employed, aiming to obtain a more accurate analysis of pre‐ and postoutbreak performance level.^14^

The fatality rate in the hospital during the outbreak was 26.9% and 30.8% for horses before discharge and the overall number of admitted horses to the VTH, respectively. These figures are within the range of fatality rates reported in previous studies (from 0%‐46%).^2^, ^8^, ^28^ This high variation in the fatality rate of EHV‐1 outbreaks is a probable consequence of the influence of a large number of factors derived from the virus, the host, and the environment that can have a direct impact on the development and severity of EHM.^3^, ^7^ The conditions surrounding sport horses, such as prolonged transport, stress, gathering of horses from different geographical regions, as well as housing conditions in competitions that do not always have optimal ventilation, make this type of horse population very susceptible to EHV‐1 outbreaks.^4^, ^5^, ^29^

The vaccination status of these horses showed a clear association with fatality. The immune system plays an important role in the development of EHM.^3^, ^26^ Repeated exposure to the virus, or even repeated immunizations, has been considered a critical factor for the development of the neurological form of the disease.^3^, ^30^ In experimental infections of horses >20 years of age, increased incidence of EHM was related to the decreased numbers of cytotoxic T‐lymphocyte (CTL) precursors.^22^ In this same study, it was concluded that critical mass reservoir of circulating memory CTLs is required for controlling EHV‐1 neurologic disease, as well as the concentration of antibodies in a horse before infection by the virus. In contrast, another study showed that EHV‐1 vaccination is an aid in decreasing clinical signs of infection and viremia, but the protection offered by a vaccine depends on the grade of exposure to EHV‐1 virus and the interval between vaccination and exposure.^30^ Results obtained in the outbreak described in our study showed that vaccination status against EHV‐1 did not improve the chances of survival or return to exercise. Vaccinated horses had higher ataxia grade and worse long‐term outcome, but our study had the limitation of not having data on the type of vaccines used or uniformity in the vaccination protocol, and consequently, further investigation is needed to draw definitive conclusions. Regardless, control of the circulating virus is important to minimize the probability of environmental exposure to the agent in the horse population. Therefore, the significant reduction in viral shedding observed in vaccinated horses provides a reasonable justification for booster vaccination of nonexposed horses.^3^

Our findings are limited by their retrospective nature and the context of a natural outbreak, which might be influenced by variables such as the population of hospitalized horses or the isolated strain among other factors. Additionally, the horses were those referred to the VTH for EHM during a large outbreak of EHV‐1, based on the severity of clinical criteria. This situation may introduce bias, because priority for admission was made for the most severely affected horses. Therefore, the results cannot necessarily be extrapolated to the general population of horses in an EHM outbreak. On the other hand, the isolated strain in this outbreak was the variant N752. Primary infection with neuropathogenic D752 results in increased nasal virus shedding in contrast with the nonneuropathogenic N752 variant. Furthermore, the D752 variant leads to higher levels of viremia and virus‐neutralizing antibody titers, inducing a stronger immune response and being more resistant to the host's response than the N752 variant.^31^ Both the higher magnitude and longer duration of cell‐associated viremia appear to contribute to the risk of development of EHM in horses infected with D752 strains of EHV‐1.^32^ The N variant has been detected more frequently in herds with abortions and less frequently in outbreaks associated with neurological signs,^6^, ^33^, ^34^ but in recent years, the relationship between the type of variant and clinical presentation has deviated from the expected results,^35^ and a concern about the virulence of the EHM variant is growing. In a recent study conducted from 2019 to 2022 in the United States,^35^ 297 swabs positives for qPCR EHV‐1 collected from horses with respiratory disease (EHV‐1), neurological disease (EHM) and abortion were analyzed for the 3 different EHV‐1 genotypes (N752, D752, and H752). The study found that the N752 genotype was the predominant variant detected in cases of EHV‐1 (87.5%), cases of EHM (74.3%) and abortions (80%). These findings are consistent with those observed in the outbreak of our study where horses developed severe EHM and the isolated virus was the N variant. Subsequent analyses involved characterization of the virus using MLST.^36^ The genetic analysis showed that the virus associated with this outbreak was closely related to other viruses circulating for several years in Europe. Special attention should be paid to the growing importance of the N variant in the development of EHM as well as its relationship with the increase in neurological disease in the last decade.

In conclusion, the fatality rate of the 2021 Valencia EHV‐1 outbreak horses admitted to this VTH was 30.8%. The main clinical variables associated with an increased risk of fatality were: (1) previous vaccination against EHV‐1; (2) presence of ataxia grade ≥4/5 on admission; and (3) development of urinary complications during the progression of the disease. Investigation of the likelihood of sport recovery of patients that overcame the disease showed that 52.9% were able to reach a similar or improved preoutbreak level of performance in show‐jumping FEI competitions within 2 years of being discharged. Ataxia grade on admission was negatively associated with the outcome of the patient. Furthermore, horses with both urinary complications and systemic signs of vasculitis were less likely to have a full sport recovery than patients with only 1 or neither of these 2 complications. Patients with grade ataxia ≥4/5 on admission had a 10% chance of performing at least to the same preoutbreak level, whereas none of the horses that developed both vascular and urinary complications returned to their previous level of performance.

## CONFLICT OF INTEREST DECLARATION

The authors declare no conflict of interest.

## INSTITUTIONAL ANIMAL CARE AND USE COMMITTEE (IACUC) OR OTHER APPROVAL DECLARATION

All owners of the horses gave informed consent for their horses to participate in the study.

## OFF‐LABEL ANTIMICROBIAL DECLARATION

The authors declare no off‐label use of antimicrobials.

## HUMAN ETHICS APPROVAL DECLARATION

The authors declare human ethics approval was not needed for this study.

## Supporting information


**Data S1.** Supporting Information.
