# Supplementary material for: Long‐term performance of show‐jumping horses and relationship with severity of ataxia and complications associated with myeloencephalopathy caused by equine herpes virus‐1
Source: J Vet Intern Med. 2024 Apr 12;38(3):1799–807. doi: 10.1111/jvim.17070 (PMC11099729; doi:10.1111/jvim.17070)
Supplement: Supplementary file 1 — Data S1. Supporting Information. [file JVIM-38-1799-s001.docx]

**Supplementary Information 1 (S1)**

**S. 1** **Therapeutic management:**

The medical therapy used in affected horses included flunixin meglumine^a^ (1.1 mg/kg IV q 24h), dexamethasone^b^ (0.1 mg/kg IV q24h for 3 days), dimethyl sulfoxide^c^ (DMSO, 1 gm/kg IV q24h for 3 days), valacyclovir^d^ (30 mg/kg every 8 hours orally lasting 7 consecutive days), diazepam^e^ (0,05 mg/kg IV) in patients with seizures. Fluid therapy based on Ringer's lactate^f^ compensated with calcium^g^ and KCl^h^ and 7.5% hypertonic saline^i^ and mannitol^j^ was administered to patients showing abnormal postures, head pressing, stupor, or compulsive walking. The fluid therapy was complemented with 5% glucose solution^k^ to ensure a correct energy balance. Lastly, omeprazole^l^ (2 mg/kg c/24h PO) and misoprostol^m^ (5 mcg/kg c/12h PO) were used as digestive ulcer prevention therapy. Horses with incontinence and cystitis were manage with trimethoprim sulfadiazine^n^ (30 mg/kg q 12 h PO), and bladder lavage using a urinary foley catheter (volume lavages between 2 and 3 Liters of physiological saline solution based on the appearance of the urine) interspersed by cystoscopy examinations.

Manufacturers’ addresses

^a^ Nixyvet 50 mg/ml solution for injection, Divasa-Farmavic, S.A., Gurb-Vic (Barcelona), ES.
^b^ Caliercortin 4 mg/ml, Laboratorios Calier, S.A., Les Franqueses del Vallès (Barcelona), ES.
^c^ Dimethyl sulfoxide, Fagron Ibérica SAU, Terrassa (Barcelona), ES.
^d^ Valacyclovir TecniGen 1000 mg tablets coated with EFG film, Tecnimede Spain Pharmaceutical Industry, S.A., Alcobendas (Madrid), ES.- * Valium
^e^ Valium 10 mg/2 ml solution for injection, Atnahs Pharma Netherlands B.V., Copenhagen, DNK.
^f^ Lactate-RingerVet, B. Braun VetCare SA, Rubí (Barcelona), ES.
^g^ Calcium Injectable Labiana, LABIANA Life Sciences, S.A., Terrassa (Barcelona), ES.
^h^ Potassium chloride 149 mg/ml (2M) Miniplasco Braun Veterinary Use, B. Braun VetCare SA, Rubí (Barcelona), ES.
^i^ Saline hypertonic 7.5 g/100 ml solution for infusion, B. Braun VetCare SA, Rubí (Barcelona), ES.
^j^ Concentrated Osmofundin 20% solution for infusion (Mannitol 200 mg/ml), B. Braun Medical SA, Rubí (Barcelona), ES.
^k^ GlucosaVet 5g/100 ml, B. Braun Vetcare SA, Rubí (Barcelona), ES.
^l^ Gastrogard 370mg/g oral paste, Boehringer Ingelheim Animal Health España, S.A.U., Sant Cugat del Vallès (Barcelona), ES.
^m^ Cytotec 200 micrograms tablets, Pfizer, S.L., Alcobendas (Madrid),ES.
^n^ Ulfaprisol, Fatro Ibérica, S.L., Sant Just Desvern (Barcelona), ES.

**Supplementary Information 2 (S2)**

**S.2 Inclusion criteria for horses in the group “clinical signs of vasculitis”**

Vasculitis is part of the pathogenesis of the EHM, but this clinical manifestation is not always visible to the clinician during the physical exam. Therefore, this study provides details on a group of animals where different clinical signs of vasculitis were evident in physical examinations. The next photography shows perineal area of a patient affected from the EHM outbreak with a systemic sign of vasculitis. The image shows congestion and petechiae in the vulvar mucosa, observed during the physical examination of one of the patients at the time of admission to the VTH, without having undergone any type of invasive procedure in the area prior to the photography.


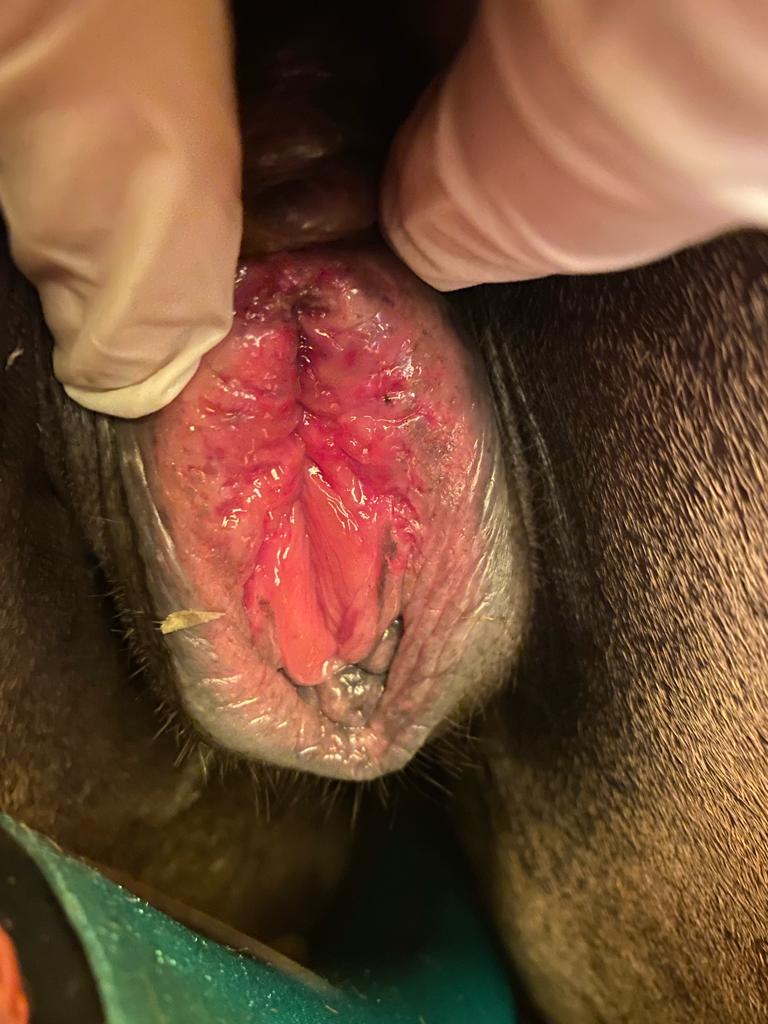


**Supplementary Information 3 (S3)**

**Physical therapy and protocol of** neuromuscular electrical stimulation (NMES)

Once the patients came out of the critical phase of the disease, rehabilitation exercises began. This therapy involved: 1) proprioception exercises based on passive stretching of the thoracic and pelvic limbs using isometric (static) exercises, such as lateral tail traction or lifting a limb, and isotonic (dynamic) exercises to promote their proprioception and coordination. The isotonic exercises consisted of hand-walking, reversal, walking on slopes or on uneven surfaces; 2) core work of abdominal and back digital pressure and lateral traction of the shoulder and croup with a raised limb to favor strength and coordination; and 3) neuromuscular electrical stimulation (NMES) was used to promote neuromuscular recovery (STIM-PRO X9+ 4CH Digital TENS/EMS). The muscle electrostimulation treatment was applied on alternate days along with core work. Electrodes were placed on the superficial gluteus and biceps femoris, with variable intensity depending on each patient's condition, muscle development, and response to initial stimuli. The program used included a stimulation mode, frequency modulation between 40-70 Hz, pulse amplitude of 300uS, on-time of 6 seconds, off-time of 12 seconds, ramp-up time of 2 seconds, ramp-down time of 1 second, for 15 minutes.

**Supplementary Information 4 (S4)**

**Supplementary Table 1. Individual clinical and sport characteristics of horses following an EHV-1 outbreak.**

|  | | |  | Agreement between  pre-outbreak and post-outbreak median performance FEI scores | | |  |  |  |  |
| --- | --- | --- | --- | --- | --- | --- | --- | --- | --- | --- |
| Horse ID | Ataxia grade on admission | Urinary complications | Systemic signs of vasculitis | Height | Ranking | Entries | Score FEI | Score  Survey | Total Score | Classification  (Full vs. Partial recovery) |
| 1 | 3 | 1 | 0 | 1 | 0 | -1 | 1 | 1 | 2 | Full |
| 2 | 1 | 1 | 0 | 1 | 1 | 1 | 1 | 1 | 2 | Full |
| 3 | 2 | 1 | 0 | 1 | 0 | 1 | 1 | 1 | 2 | Full |
| 4 | 3 | 0 | 1 | 1 | 0 | 1 | 1 | 1 | 2 | Full |
| 5 | 0 | 0 | 0 | 1 | 0 | 1 | 1 | 1 | 2 | Full |
| 6 | 3 | 0 | 1 | 0 | 0 | 0 | 1 | 1 | 2 | Full |
| 7 | 4 | 0 | 1 | 0 | 0 | -1 | 1 | 1 | 2 | Full |
| 8 | 0 | 0 | 0 | 0 | -1 | 0 | 1 | 1 | 2 | Full |
| 9 | 3 | 0 | 0 | No FEI Data post outbreak | | |  | -1 | -1 | Partial |
| 10 | 3 | 1 | 1 | 0 | 0 | -1 | 1 | -1 | 0 | Partial |
| 11 | 3 | 0 | 1 | 0 | 0 | 0 | 1 | -1 | 0 | Partial |
| 12 | 4 | 1 | 1 | 0 | 0 | 0 | 1 | -1 | 0 | Partial |
| 13 | 3 | 1 | 0 | 1 | 1 | 1 | 1 | 1 | 2 | Full |
| 14 | 3 | 1 | 1 | 1 | 0 | 0 | 1 | -1 | 0 | Partial |
| 15 | 4 | 1 | 1 | No FEI Data post outbreak | | |  | -1 | -1 | Partial |
| 16 | 4 | 1 | 1 | -1 | 1 | -1 | -1 | -1 | -2 | Partial |
| 17 | 1 | 0 | 0 | -1 | 1 | 1 | 1 | -1 | 0 | Partial |
